# CD4 T Cell Immunity Is Critical for the Control of Simian Varicella Virus Infection in a Nonhuman Primate Model of VZV Infection

**DOI:** 10.1371/journal.ppat.1002367

**Published:** 2011-11-10

**Authors:** Kristen Haberthur, Flora Engelmann, Byng Park, Alex Barron, Alfred Legasse, Jesse Dewane, Miranda Fischer, Amelia Kerns, Monica Brown, Ilhem Messaoudi

**Affiliations:** 1 Department of Molecular Microbiology and Immunology, Oregon Health and Science University, Portland, Oregon, United States of America; 2 Vaccine and Gene Therapy Institute, Oregon Health and Science University, Beaverton, Oregon, United States of America; 3 Division of Biostatistics, Department of Public Health and Preventive Medicine, Oregon Health and Science University, Portland, Oregon, United States of America; 4 Division of Pathobiology and Immunology, Oregon National Primate Research Center, Beaverton, Oregon, United States of America; University of Alabama at Birmingham, United States of America

## Abstract

Primary infection with varicella zoster virus (VZV) results in varicella (more commonly known as chickenpox) after which VZV establishes latency in sensory ganglia. VZV can reactivate to cause herpes zoster (shingles), a debilitating disease that affects one million individuals in the US alone annually. Current vaccines against varicella (Varivax) and herpes zoster (Zostavax) are not 100% efficacious. Specifically, studies have shown that 1 dose of varivax can lead to breakthrough varicella, albeit rarely, in children and a 2-dose regimen is now recommended. Similarly, although Zostavax results in a 50% reduction in HZ cases, a significant number of recipients remain at risk. To design more efficacious vaccines, we need a better understanding of the immune response to VZV. Clinical observations suggest that T cell immunity plays a more critical role in the protection against VZV primary infection and reactivation. However, no studies to date have directly tested this hypothesis due to the scarcity of animal models that recapitulate the immune response to VZV. We have recently shown that SVV infection of rhesus macaques models the hallmarks of primary VZV infection in children. In this study, we used this model to experimentally determine the role of CD4, CD8 and B cell responses in the resolution of primary SVV infection in unvaccinated animals. Data presented in this manuscript show that while CD20 depletion leads to a significant delay and decrease in the antibody response to SVV, loss of B cells does not alter the severity of varicella or the kinetics/magnitude of the T cell response. Loss of CD8 T cells resulted in slightly higher viral loads and prolonged viremia. In contrast, CD4 depletion led to higher viral loads, prolonged viremia and disseminated varicella. CD4 depleted animals also had delayed and reduced antibody and CD8 T cell responses. These results are similar to clinical observations that children with agammaglobulinemia have uncomplicated varicella whereas children with T cell deficiencies are at increased risk of progressive varicella with significant complications. Moreover, our studies indicate that CD4 T cell responses to SVV play a more critical role than antibody or CD8 T cell responses in the control of primary SVV infection and suggest that one potential mechanism for enhancing the efficacy of VZV vaccines is by eliciting robust CD4 T cell responses.

## Author Summary

Varicella zoster virus (VZV) causes chickenpox and establishes a life-long latent infection in humans. VZV can reactivate years later to cause shingles, a debilitating and painful disease. Vaccines against both chickenpox and shingles are available but not 100% efficacious. Two doses of the chickenpox vaccine are required to provide adequate protection and the shingles vaccine reduces the incidence of this disease by 51%. To improve these vaccines, we must identify the components of the immune system that are important for the control of VZV replication. However, the contribution of T versus B cell responses is unknown. Infection of rhesus macaques with simian varicella virus is a robust model of VZV infection. Here, we used this unique animal model to show for the first time that the absence of B cells does not alter disease severity and that the loss of CD8 T cells only results in a mild increase in disease severity. In sharp contrast, the lack of CD4 T cells leads to disseminated varicella. These data highlight the importance of CD4 T cells and suggest that novel vaccines that focus on engendering a more robust CD4 T cell response against VZV might provide better protection from chickenpox and shingles.

## Introduction

Varicella zoster virus (VZV), a neurotropic alphaherpesvirus, is the causative agent of varicella (chickenpox). Following resolution of the acute infection, VZV establishes latency in sensory ganglia, and can reactivate years later, manifesting as dermatomal vesicular lesions known as herpes zoster (HZ, shingles) [1]. HZ is a painful and debilitating disease that causes significant morbidity such as post-herpetic neuralgia and HZ opthalmicus [2], [3] and occasionally mortality in the elderly and immune compromised [4]. HZ affects 1 million people each year in the United States [5], [6] and persons older than 60 year of age account for 40–50% of HZ cases reported each year [5], [6]. Given that by 2020 17% of the US population is estimated to be 65 years of age or older (US Census), the incidence of HZ and its associated morbidities is likely to increase.

There are currently two FDA approved VZV vaccines available that contain the live attenuated VZV Oka strain: Varivax, directed against chickenpox, and Zostavax, directed against shingles. The introduction of Varivax, and more specifically of the 2-dose regimen, has dramatically reduced the incidence of chickenpox and annual varicella-related hospitalizations and deaths in the US [7], [8]. Similarly, vaccination with Zostavax reduced the incidence of shingles by 51% in a 3-year study period and resulted in a 61% decrease in the burden of disease [9], [10]. However, it is important to note that the efficacy of Zostavax was reduced in individuals older than 70 years [9]. Moreover, recent studies showed that the cellular and humoral responses engendered by Zostavax significantly declined 2 years following Zostavax vaccination [11]. Given that the proportion of persons 85 years and older is expected to increase from 14% in 2010 to more than 21% in 2050 (US Census Bureau, 2010), new vaccination strategies against VZV should be explored.

Clinical observations suggest that the successful resolution of acute VZV infection is associated with the development of the host's VZV-specific cellular immunity rather than humoral immunity [1], [12], [13], [14], [15], [16]. Specifically, children suffering from congenital immune deficiencies affecting cellular immunity or from hematological malignancies, or undergoing immunosuppressive treatment are at risk for progressive varicella; whereas, children with agammaglobulinemia have uncomplicated varicella episodes [17], [18], [19], [20], [21]. Transfusion of lymphocytes from immune donors to children with immunodeficiencies can limit VZV infection and replication after the appearance of cutaneous lesions [22]. In contrast, the administration of immunoglobulins with high titers of IgG antibodies to VZV is only protective when administered within 72 hours of exposure [23], [24], [25]. While early production of VZV antibodies by HIV-infected children does not prevent progressive varicella [26], absence of cellular immunity in these children correlates with a risk of prolonged viremic phase, continued formation of skin lesions and dissemination of the virus to the lungs and other organs [16], [27]. However, these data are confounded by the fact that T cell-deficient patients lack both T cell-mediated and T cell-dependent B cell-mediated responses. Thus, more careful analyses of the contribution of each of the major lymphocyte subsets are required.

Both VZV-specific CD4 and CD8 T cells are detected during acute VZV infection [12], [28]. CD4 Th1 cells, which produce the anti-viral cytokine IFNγ and have cytotoxic potential, predominate the VZV-specific T cell response [12], [28], with little or no production of Th2 cytokines [12]. Several studies have shown that VZV-specific CD4 T cells recognize ORF4 [29], glycoprotein I [30], and ORF63 [31]. In contrast to CD4 T cells, the specificity and magnitude of VZV-specific CD8 T cell response are only partially understood. Several studies have suggested that VZV-specific CD8 T cells circulate at very low frequencies, possibly due to immune evasion strategies such as MHC class I downregulation [30], [32], [33]. However, it is also likely that the CD8 T cell response is under-appreciated because VZV lysate used in the majority of the studies is poorly presented via the MHC-I pathway [34]. Consequently, the role of CD8 versus CD4 T cell immunity in the control of VZV infection remains unclear.

Our understanding of the importance of the T and B cell responses to the resolution of VZV infection remains incomplete because of the lack of adequate animal models. VZV infection of small rodent models (guinea pigs, cotton rats, and mice) does not result in varicella [35], [36], [37], [38], [39]. Dr. Arvin and colleagues developed a SCID-humanized (SCID-hu) mouse model which has significantly advanced our understanding of VZV pathogenesis [40]. However, the immuno-deficient nature of the animals has precluded the characterization of immune response to VZV infection. Simian varicella virus (SVV) is a neurotropic alphaherpesvirus that naturally infects non-human primates (NHP) and shares 75% DNA homology and genome colinearity with VZV [41], [42], [43]. The exact mode of SVV transmission has not yet been experimentally determined but it is believed to occur through contact with skin lesions of an infected animal, or via exposure to virus-laden aerosolized droplets from infected animal(s), as described for VZV transmission in humans [44], [45]. We have recently shown that intrabronchial infection of rhesus macaques (RM) with SVV recapitulates the hallmarks of acute VZV infection in humans: (1) generalized varicella rash; (2) development of cellular and humoral responses; (3) resolution of acute infection; and (4) establishment of latency in sensory ganglia [46]. In our model, the introduction of SVV directly into the lungs potentially bypasses the initial stage of VZV replication that occurs in the oropharynx, head and neck regions [47], which could affect viral amplification within tonsilar memory T cell [40], [48], [49]. Despite this potential difference, T and B cell responses develop with similar kinetics relative to the onset of the exanthem in SVV inoculated animals and children infected with VZV, thereby providing a robust animal model with which to study anti-VZV immunity.

The goal of this study was to identify the host immune responses essential for protection against primary SVV infection in unvaccinated RM as a model of VZV infection in humans. To this end, we compared disease severity and immune response in four groups of young RM infected with SVV: (1) control animals; (2) CD20+ B cell depleted; (3) CD8+ T cell depleted; and (4) CD4+ T cell depleted. We show that loss of B cells during primary SVV infection does not alter viral loads or disease severity. In contrast, loss of CD8 T cells led to a slightly higher peak viral load and the loss of CD4 T cells led to significantly higher viral loads and disseminated varicella. These studies suggest that cellular immunity and more specifically CD4 T cell immunity plays a critical role in the control of SVV infection. These observations have important ramifications for the development of novel vaccination strategies to alleviate VZV associated diseases.

## Results

### Efficacy of depleting antibodies

**Figure 1:**
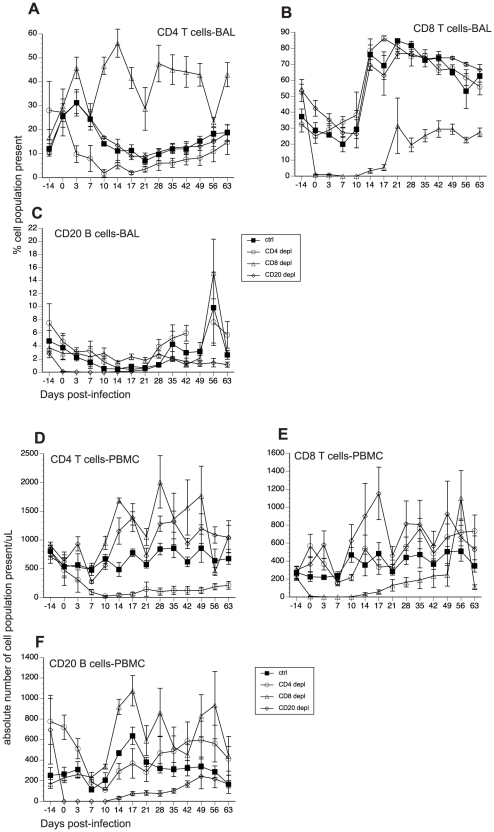
Efficacy of antibody-mediated depletion of immune cells during acute SVV infection. (A–C) Average frequency of CD4 T (A), CD8 T (B), and CD20 B (C) cells in bronchial alveolar lavage (BAL) of control, CD20 depleted, CD8 depleted, and CD4 depleted animals (n = 4/group) were measured using flow cytometry (FCM). Absolute numbers per µl/blood were then calculated by converting the percentage of these subsets using complete blood counts obtained at every time point (D–F).

The contributions of cellular versus humoral immune responses in the resolution of acute VZV infection have not yet been experimentally addressed. Therefore, using the infection of young RM with SVV as a model of acute VZV infection, we aimed to determine the role of T cell versus B cell responses during acute SVV infection. Prior to intrabronchial SVV inoculation, 16 young RM were divided into four groups of four animals each: (1) control; (2) CD8+ T cell depleted; (3) CD4+ T cell depleted; and (4) CD20+ B cell depleted. T and B cell depletion regimens were initiated 7 days prior to infection to ensure that the targeted lymphocyte population was not present on the day of infection (0 dpi). The frequency of CD4+ T cells, CD8+ T cells, and CD20+ B cells in peripheral blood mononuclear cells (PBMC) and bronchial alveolar lavage (BAL) samples were monitored throughout the study by flow cytometry (FCM). In contrast to peripheral blood, frequency of CD20 B cells is very low in BAL (Fig. 1). Treatment with CD20 depleting antibody resulted in the loss of B cells beginning at 0 dpi and lasting until 21 dpi in BAL (Fig. 1C) and 17 dpi in peripheral blood (Fig. 1F). Administration of CD8-depleting antibody resulted in complete loss of CD8+ T cells between 0 and 14 dpi in BAL (Fig. 1B) and between 0 and 17 dpi in peripheral blood (Fig. 1E) Administration of anti-CD4 depleting antibody decreased the frequency of CD4+ T cells on day 0 but complete loss was not achieved until 10 dpi (Fig 1A and D). This loss was very transient in BAL samples where the recovery started 14 dpi (Fig. 1A). In peripheral blood, CD4 depletion lasted until 17 dpi (Fig. 1D). In summary, CD20 and CD8 T cell depletions were more profound (achieving ∼100% loss by day 0) and lasted longer than CD4 T cell depletion. Following depletion, recovery was very slow in all three lymphocyte subsets and the frequencies did not return to baseline by the end of the study.

### Impact of T and B cell loss on SVV viral loads and disease severity

**Figure 2:**
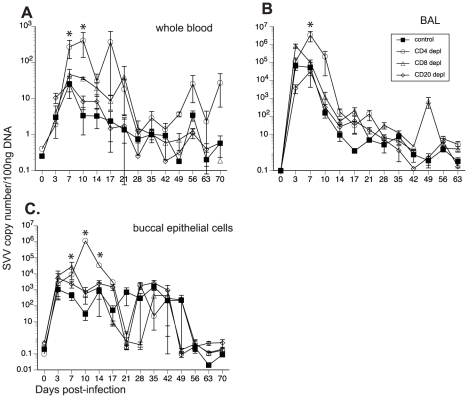
Detection of SVV viral loads in control and depleted animals. SVV viral DNA loads were assessed by quantitative real-time PCR in (A) whole blood (WB), (B) bronchial alveolar lavage (BAL), and (C) buccal epithelial cell samples from control, CD20 depleted, CD8 depleted, and CD4 depleted animals (n = 4/group). Averages± standard error of the mean (SEM) are shown. * indicates p<0.05 as compared to control animals.

**Figure 3:**
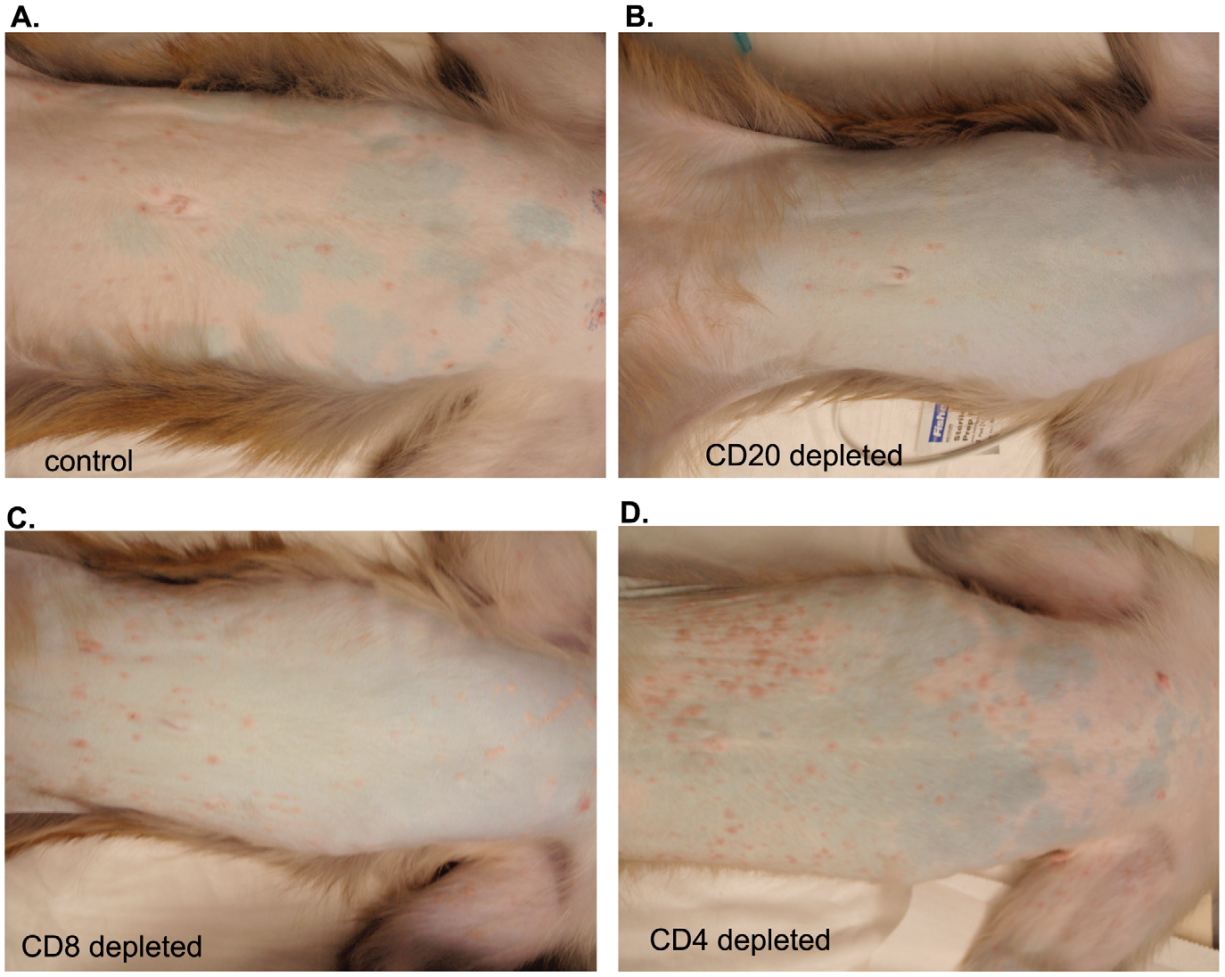
Representative examples of varicella in control and depleted animals. Varicella rash on the trunk of a representative (A) control, (B) CD20 depleted, (C) CD8 depleted, and (D) CD4 depleted animal at 10 dpi.

To determine the impact of T and B cell loss on SVV replication, we measured viral loads in whole blood (WB), BAL and buccal swabs by qPCR. As previously reported [46], SVV viral loads in WB were detected in non-depleted control animals 3 dpi, peaked at 7 dpi, and resolved at 21 dpi in WB (Fig. 2A) and BAL (Fig. 2B). CD20 depleted animals showed comparable viral loads and viral replication kinetics as those observed in non-depleted animals (Fig. 2A and 2B). In contrast, CD4+ depleted animals showed the highest peak SVV viral loads in WB at 7 dpi (p = 0.05) and 10 dpi (p = 0.002) (Fig. 2A) and BAL at 7 dpi (p<0.001) (Fig. 2B) compared to non-depleted control animals. They also showed recurrent viremia as evidenced by the detection of SVV viral DNA in WB on days 56 and 70 (Fig. 2A). CD8 depleted animals also experienced higher viral loads 7 dpi in WB (Fig. 2A) and 3 dpi in the BAL (Fig. 2B) than non-depleted animals but these differences did not reach statistical significance. We also measured SVV shedding in saliva since it is well documented that VZV reactivation results in the shedding of infectious VZV in the saliva [50], [51]. SVV viral DNA was only detected in the buccal epithelial cells and not the saliva (Fig. 2C and data not shown). CD8 depleted animals experienced higher SVV viral loads in buccal epithelial cells at 7 dpi (p = 0.0418), while CD4 depleted animals showed higher SVV viral loads in buccal epithelial cells at 10 (p<0.001) and 14 dpi (p = 0.0182) in comparison to non-depleted control animals (Fig. 2C).

All 16 animals developed varicella (Fig. 3), but the severity and duration of the rash varied between groups (Table 1). As previously reported [46], control animals developed lesions beginning at 7 dpi that began to heal 14 dpi and were completely resolved 21 dpi (Table 1; Fig. 3A). CD20 depleted animals had comparable disease severity to control animals (Fig. 3B). However, CD8 depleted animals continued to display moderate to severe lesions at 14 dpi that did not resolve until 28 dpi (Table 1; Fig. 3C). CD4 depleted animals experienced severe and disseminated varicella rash (Fig. 3D), which also did not begin to heal until 42 dpi (Table 1). Taken together, these data show that in contrast to CD20 B cells, the absence of CD8 or CD4 T cells results in higher viral loads and increased disease severity during primary SVV infection.

**Table 1:** Summary of rash duration, lesion number, and disease severity of control and experimental animal groups following acute SVV infection.

Group	Rash duration	Number of lesions	Disease severity
Control	10–14 days	∼40	Mild
CD20 depleted	10–14 days	∼40	Mild
CD8 depleted	18–21 days	>125	Moderate-severe
CD4 depleted	25–28 days	>200	severe

The number of lesions for each group was determined by averaging the lesions observed on the abdomen of each animal within each group at 10dpi. The disease severity of each group was determined by averaging the assigned severity score (based on the size and duration of observed vesicles) of each animal within each group (1–2 = mild; 3–4 = moderate; 5 = severe).

### Modulation of B cell response by T and B cell depletion

**Figure 4:**
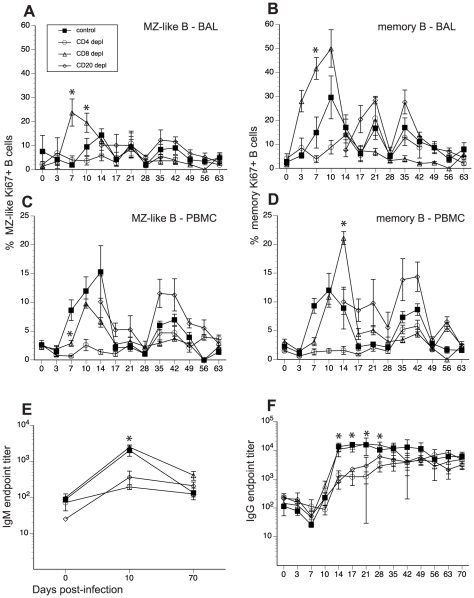
Impact of T and B cell depletion on kinetics and magnitude of B cell proliferation and IgG/IgM production following SVV infection. Frequency of proliferating (Ki67+) B cells within marginal zone-like and memory subsets in BAL (A, B) and PBMC (C, D) was measured using FCM. Data points for B cell proliferation in CD20-depleted animals are not shown 0–14 dpi as there were no B cells in circulation during this time period. Average SVV-specific IgM (E) and IgG (F) end point titers± SEM in control, CD20 depleted, CD8 depleted, and CD4 depleted animals (n = 4/group) were determined by standard ELISA.* indicates p<0.05 as compared to control animals.

We next investigated the impact of lymphocyte depletion on the proliferative response of B cells and the generation of SVV-specific IgG, IgM, and neutralizing antibody responses. Following antigen encounter, naïve B cells undergo a proliferative burst and acquire the memory marker CD27. Therefore, we assessed the proliferative response of B cells by measuring changes in the expression of Ki67, a nuclear protein associated with G2-S cell cycle transition, within two major antigen-experienced B cell subsets: marginal zone-like (MZ-like) and memory B cell subsets, using FCM. In BAL from control animals, proliferation of MZ-like B cells began 10 dpi, peaked 14 dpi, and returned to baseline levels 17 dpi (Fig. 4A). Frequency of Ki67+ memory B cells in BAL of control animals increased at 7dpi, peaked 10dpi, and returned to baseline levels 17 dpi (Fig. 4B). In CD20-depleted animals, Ki67+ MZ-like and memory B cell in BAL were detected 14 dpi as the B cell compartment began to regenerate (Fig. 4A and 4B). Interestingly, we detected an earlier onset of proliferation of MZ-like and memory B cells in BAL in CD8 depleted animals compared to control animals (Fig. 4A and 4B). Proliferative burst of the MZ-like B cells peaked 7 days before control animals (7 dpi versus 14 dpi) and reached a higher level (p<0.0001) before returning to baseline levels 17 dpi (Fig. 4A). Similarly, the proliferative burst of the memory B cells was detected 4 days earlier (3dpi versus 7dpi) and reached a higher peak 7 dpi compared to control animals (p = 0.0003) (Fig. 4B). In contrast, we detected minimal B cell proliferation with no distinct kinetics (less than 10%) in both the MZ-like and memory subsets in CD4 depleted animals (Fig. 4A and 4B, and Table 2).

**Table 2:** Summary of effect of immune cell depletions on the anti-SVV response.

	Control animals	CD20 depleted animals	CD8 depleted animals	CD4 depleted animals
B cell proliferation	Peaked at 10–14 dpi	N/A	Earlier onset, higher magnitude	Delayed onset, significantly reduced magnitude
CD4 T cell proliferation	Peaked at 7 dpi	Similar to control animals	Similar kinetics, higher magnitude	N/A
CD8 T cell proliferation	Peaked at 7 dpi	Similar to control animals	N/A	Delayed onset, significantly reduced magnitude
Frequency of SVV-specific CD4 T cell	Peaked at 7 dpi	Lower frequency at 7/14 dpi	Lower frequency at 14 dpi in BAL	N/A
Frequency of SVV-specific CD8 T cell	Peaked at 7 dpi	Lower frequency at 7/14 dpi	N/A	Lower frequency at 7/14 dpi
CD4 grzmB+ T cell	Peaked at 3–7 dpi	Significantly lower magnitude at 7 dpi in CD4 CM PBMC	Significantly lower magnitude at 7 dpi in CD4 CM PBMC	N/A
CD8 grzmB+ T cell	Peaked at 7 dpi	Similar to control animals	N/A	Significantly lower magnitude at 7 dpi in CD4 CM in BAL & PBMC

Hallmarks of the immune response during acute SVV infection were compared between control and depleted animals. N/A indicates that comparison could not be carried out due to the depletion of the T or B cell subset in question.

In PBMCs of control animals, MZ-like and memory B cell proliferation was detected 7 dpi, peaked 10–14 dpi, before returning to baseline levels 17 dpi (Fig. 4C and 4D). As described for BAL, Ki67+ MZ-like and memory B cells were detected 14 dpi in CD20 depleted animals (Fig. 4C and 4D), coinciding with the re-appearance of B cells in peripheral blood (Fig. 1). In CD8 depleted animals the proliferative burst of MZ-like and memory B cells was delayed by 3 days (10 dpi compared to 7 dpi, p = 0.008) (Fig. 4C and 4D) but reached higher peak value 14 dpi in the memory subset compared to control animals (p<0.001) (Fig. 4D). As observed in BAL, B cells from CD4 depleted animals showed minimal proliferation in response to SVV infection (p<0.05 at all time points examined between 7 and 14 dpi) (Fig. 4C and 4D, and Table 2).

To assess differences in the magnitude of the proliferative burst, we measured the area under the curve (AUC) for MZ-like and memory B cell proliferation between 3 and 17 dpi. This analysis revealed that the magnitude of the proliferative burst of MZ-like and memory B cells was significantly reduced in PBMC in CD4 depleted animals compared to control animals (p<0.001). In CD8 depleted animals, memory and MZ-like B cell proliferative burst was higher in BAL (p = 0.025) but MZ-like proliferation was lower in PBMC compared to controls (p = 0.013) (Table 2).

We also measured the impact of T and B cell depletion on antibody production. To that end, we measured SVV-specific IgM and IgG titers using standard ELISA and neutralizing antibody titer using plaque reduction assay. In control and CD8 depleted animals, SVV-specific IgM titers peaked 10 dpi (average titer 1∶2000 and 1∶2465, respectively) (Fig. 4E). In contrast, CD20 and CD4 depleted animals had significantly lower average titers of 1∶360 and 1∶190, respectively (Fig. 4E, p<0.05). Similarly, control and CD8 depleted animals generated a robust SVV-specific IgG response that peaked 21 dpi (average titer 1∶15800 and 1∶17300 respectively) (Fig. 4F), whereas CD20 and CD4 depleted animals had significantly reduced IgG antibody production 14, 17, 21, and 28 dpi compared to control animals (p<0.02 for all time points). Specifically, SVV-specific IgG titers 21 dpi were on average 1∶6200 and1∶2500 in CD20 and CD4 depleted animals respectively (Fig. 4F). Analysis of the AUC for SVV-specific IgG titers between 0 and 63 dpi showed that both CD20 depleted and CD4 depleted animals have significantly reduced IgG production compared to non-depleted control animals (p = 0.0245 and p = 0.0193, respectively) (Fig. 4F).

We also assessed neutralizing antibody titers following acute SVV infection 14 and 70 dpi in all animal groups by measuring the dilution at which 50% reduction in the number of SVV plaques was achieved (NT50, Table 3). NT50 titers were comparable between control and CD8 depleted animals 14 dpi, and by 70 dpi the titers increased 34- to 90-fold and 8- to 17-fold, respectively (Table 3). In contrast, CD4 depleted animals had considerably lower NT50 titers 14 dpi compared to control animals, which increased 6- to 16-fold by 70 dpi (Table 3). Plasma obtained from CD20 depleted animals showed no neutralization ability 14 dpi and very negligible NT50 titers 70 dpi (Table 3). These data suggest that the resolution of varicella during primary SVV infection in unvaccinated animals does not require the presence of neutralizing antibodies.

**Table 3:** Neutralizing antibody response following acute SVV infection.

Animal I.D.	Group	14 dpi	70 dpi
24943	control	42	53
25170	control	24	2178
25339	control	280	9564
27577	control	177	9862
25152	CD4 depl	21	348
25111	CD4 depl	ND	481
24952	CD4 depl	ND	1869
24993	CD4 depl	9	119
25343	CD8 depl	22	381
25371	CD8 depl	89	855
26842	CD8 depl	49	456
26108	CD8 depl	121	975
25833	CD20 depl	ND	60
25905	CD20 depl	ND	20
25920	CD20 depl	ND	33
25892	CD20 depl	ND	65

Neutralization titers are expressed as the reciprocal of the plasma dilution that neutralized 50% or more of the plaques compared to control cultures (no plasma added). Neutralization titers less than the starting dilution of 1∶2 are listed as ND (not determined).

### Modulation of T cell response by B and T cell depletion

**Figure 5:**
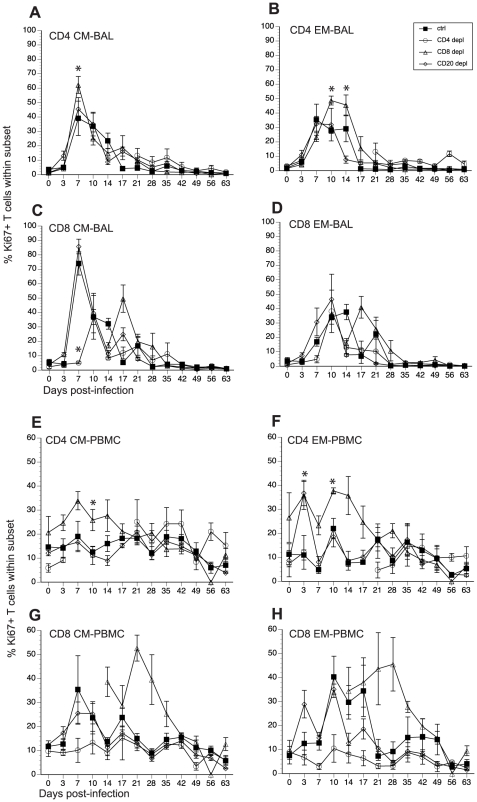
Impact of T and B cell depletion on the kinetics and magnitude of T cell proliferation following acute SVV infection. Frequency of proliferating (Ki67+) CD4 and CD8 T cells within central and effector memory subsets was measured in BAL (A–D) and PBMC (E–H) by FCM. Data points for CD4 T cell Ki67+ frequency in CD4-depleted animals are not shown 7–21 dpi as there were no CD4 T cells in circulation during this time period. Similarly data points for Ki67+ CD8 T cell frequency are not shown 0–14 dpi as there were no CD8 T cells detected during this period. * indicates p<0.05 as compared to control animals.

We assessed how loss of T and B cells modulated the kinetics and magnitude of T cell proliferation in BAL and PBMCs in response to SVV infection. Like B cell responses, after antigen encounter, naïve T cells become activated, differentiate into central and effector memory (CM and EM) T cells and undergo a robust proliferative burst. We assessed this proliferative burst by measuring frequency of Ki67+ cells within CM and EM T cell subsets in BAL and PBMC [46]. In BAL from control animals, T cell proliferation was detected in all subsets 7 dpi after which the frequency of Ki67+ T cells gradually declined reaching baseline levels 17 dpi (Fig. 5A–D, and Table 2). In CD20 depleted animals, the kinetics and magnitude of proliferation in all four T cell subsets was similar to that observed in control animals (Fig. 5A–D). The kinetics of the CD4 T cell proliferation in CD8 depleted animals was comparable to that observed in control animals. However, the peak frequency of Ki67+ CD4 CM at 7 dpi for CD8 depleted animals was significantly larger than that observed for control animals (p = 0.0028) (Fig. 5A). Similarly, the peak frequency of Ki67+ CD4 EM T cells 10 dpi and 14 dpi was also higher in CD8 depleted animals (p = 0.0054, p = 0.0058, respectively) (Fig. 5B). Interestingly the re-appearance of CD4 T cells in the BAL 21 dpi was not accompanied by an increased frequency of CD4 Ki67+ T cells (Fig. 5A and 5B). We detected proliferating CD8 T cells in CD8 depleted animals 17 dpi (Fig. 5C and 5D), which coincides with the re-appearance of CD8 T cells in these animals (Fig. 1). CD8 CM proliferation was significantly delayed (10 dpi versus 7 dpi in control animals) and reduced in CD4 depleted animals (p<0.001 at 7 dpi; p = 0.0059 at 14 dpi) (Fig. 5C). Similarly, proliferation of CD8 EM in CD4 depleted animals was also delayed (10 dpi versus 7 dpi in control animals, Fig. 5D).

We detected very little proliferation by the CD4 T cells in PBMC from control animals (Fig. 5E and 5F, and Table 2). On the other hand, CD8 T cell proliferation was easily detected in PBMC in control animals (Fig. 5G and 5H, and Table 2). The frequency of Ki67+ CD8 CM T cells in control animals peaked 7 dpi and returned to baseline 14 dpi, and the frequency of Ki67+ CD8 EM T cells peaked 10 dpi before returning to baseline levels 21 dpi (Fig 5G and 5H). The kinetics and magnitude of T cell proliferative burst in CD20 depleted animals were comparable to those described for control animals (Fig. 5E–H). As described for the BAL, CD4 T cell proliferation was more robust in CD8 depleted animals compared to controls. Specifically, the frequency of Ki67+ CD4 CM T cells was significantly higher 10 dpi (p = 0.0138) (Fig. 5E), and that of CD4 EM T cells was significantly higher 3, 7, 10, 14, and 17 dpi (p<0.01 for all days) (Fig. 5F) when compared to control animals. Ki67+ CD8 CM and EM T cells were detected 14 dpi (Fig. 5G and H), which coincided with the re-appearance of CD8 T cells (Fig. 1). As described for the BAL, CD4 depleted animals exhibited a significant reduction of proliferating CD8 CM (p = 0.001) and EM (p< 0.01 for 10,14 and 17 dpi) (Fig. 5G and 5H). As previously noted for the BAL, regeneration of the CD4 T cell compartment was not accompanied by an increase in CD4 T cell proliferation in these animals (Fig. 5G and 5H).

As described above for B cells, we measured the AUC from 3 to 17 dpi for T cell proliferation to assess the magnitude of the proliferative burst. This analysis showed that in BAL the proliferative burst of: 1) CD4 EM T cells was significantly higher in CD8 depleted animals (p = 0.026) and 2) that of CD8 CM was significantly lower in CD4 depleted animals (p<0.001) compared to control animals. In peripheral blood: 1) CD8 depleted animals experienced a larger proliferative burst in the CD4 CM (p = 0.08) and EM (p <0.001) compared to controls and 2) the CD4 depleted animals showed a smaller CD8 CM (p = 0.02) and EM (p <0.001) T cell proliferative burst compared to control animals (Table 3).

### Effect of T and B cell depletions on responding T cell frequency

**Figure 6:**
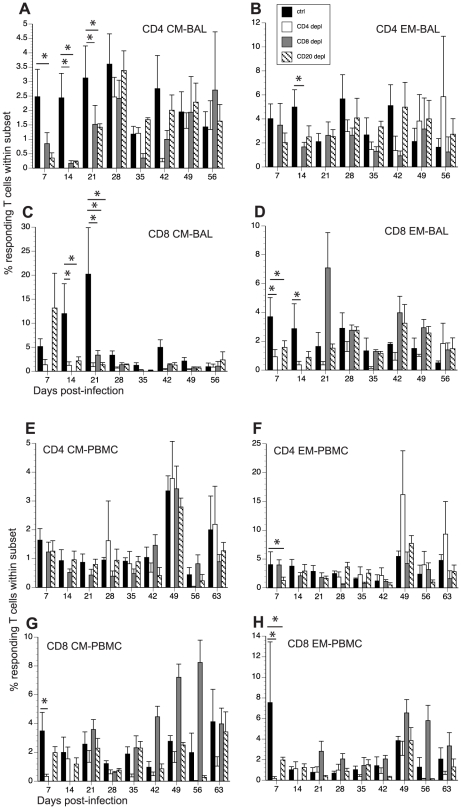
Impact of T and B cell depletion on frequency of SVV-specific T cells. The frequency of SVV-specific T cells in BAL and PBMCs was measured by intracellular cytokine staining following stimulation with overlapping peptide pools covering ORFs 4, 31, 61 and 63. The average percentage of responding (IFNγ+ and IFNγ+TNFα+) T cells ± SEM within CD4 CM, CD4 EM, CD8 CM and CD8 EM subsets in BAL (A–D) and PBMCs (E–H) is shown. Responses detected on day 0 were on average <0.5% and were subtracted from subsequent time points. * indicates p<0.05 as compared to control animals.

In addition to comparing the kinetics and magnitude of the T cell proliferative burst in response to SVV in the different groups, we determined the frequency of SVV-specific T cells within CM and EM subsets by measuring frequency of IFNγ+ or IFNγ+/TNFα+ following stimulation of PBMC and BAL cells with overlapping peptide pools that covered SVV ORFs 4, 31, 61, and 63 using intracellular cytokine staining (ICS) (Fig. 6, and Table 2).

**Figure 7:**
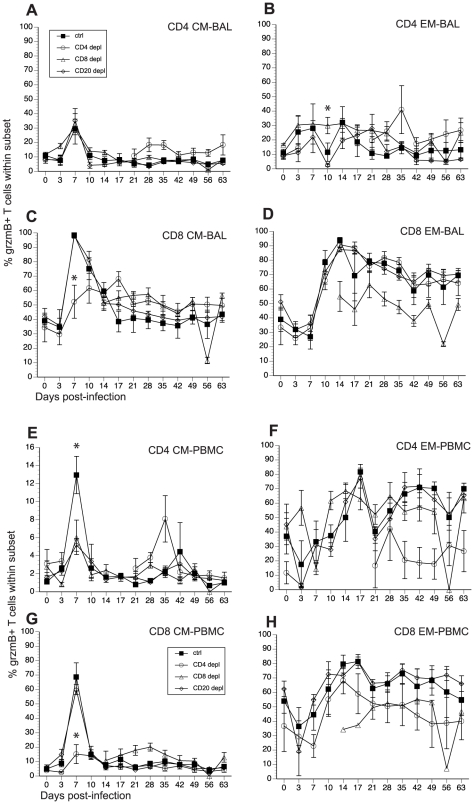
Frequency of granzyme (grzm) B+ T cells after SVV infection. Frequency of grzmB+ T cells within CD4 and CD8 CM and EM subset was assessed by FCM in BAL (A–D) and PBMC (E–H). Average percentages of grzmB+ cells ± SEM are shown. Data points for CD4 T cell grzmB+ frequency in CD4-depleted animals are not shown 7–21 dpi as the frequency of CD4 T cells in circulation was too low during this time period. Similarly, data points for grzmB+ CD8 T cell frequency are not shown 0–14 dpi as there were no CD8 T cells detected during this period. * indicates p<0.05 as compared to control animals.

Following stimulation with overlapping peptide pools, SVV-specific CD4 CM and EM T cells in BAL were detected at 7 dpi in control animals, and their frequencies remained stable until 56 dpi (Fig. 6A and 6B). SVV-specific CD8 CM and EM T cells were also detected 7 dpi. Whereas the frequency of SVV-specific CD8 CM T cells increased from 7 to 21 dpi after which it declined, that of CD8 EM T cells remained stable until 56 dpi (Fig. 6C and D). Frequency of SVV-specific CD4 CM T cells were lower in BAL from CD20 depleted animals 7–21 dpi compared to control animals (p = 0.01, 0.008 and 0.03 respectively, Fig. 6A). The frequencies of SVV-specific CD8 CM in BAL were initially comparable between CD20 depleted animals and controls but decayed faster in CD20 depleted animals 21 dpi (p<0.001, Fig. 6C). The frequency of SVV-specific CD8 EM was also initially lower in BAL in CD20 depleted animals 7 and 14 dpi (0.03 and 0.04 respectively, Fig. 7D). After day 21, frequency of SVV-specific T cells was comparable in CD20 depleted and control animals. In CD8 depleted animals the frequency of responding T cells within CD4 CM subset was lower than that detected in control animals 7–21 dpi (p = 0.04, p = 0.006 and p = 0.04 respectively, Fig. 6A). Similarly the frequency of responding T cells within CD4 EM was lower 14 dpi compared to controls (p = 0.0413) (Fig. 6B). SVV-specific CD8 T cells were first detected in CD8 depleted animals 21 dpi, which corresponds to the re-appearance of the CD8 T cells in these animals, albeit with lower frequency within the CD8 CM subset compared to control animals (p<0.001, Fig. 6C). SVV-specific CD4 T cells appeared in CD4 depleted animals 28 dpi and their frequencies within the CM and EM subsets were comparable to those observed in control animals. It should be noted that 21–28 dpi, the number of CD8 and CD4 T cells is still much lower in CD8 and CD4 T cell depleted animals respectively compared to controls. Therefore, although the relative frequencies of responding T cells within CM and EM subsets may be comparable, the absolute number of SVV specific CD8 and CD4 T cells is much lower in CD8 and CD4 depleted animals respectively compared to controls. In contrast, frequency of SVV-specific CD8 T cells within CM and EM subsets in CD4 depleted animals were lower than those observed in control animals at several time points (14 and 21 dpi in CD8 CM p = 0.007 and <0.001, respectively; and 7 and 14 dpi in CD8 EM p = 0.02 and 0.01, respectively, Fig. 6C and 6D).

In PBMCs, SVV-specific T cells were detected 7 dpi in control animals and their frequency remained stable until 56 dpi (Fig. 6E–H). In CD20 depleted animals, SVV-specific T cells were also detected 7 dpi, but the frequencies of CD4 EM and CD8 EM were lower compared to control animals (p = 0.03 and p = 0.007, respectively) (Fig. 6E–H). SVV-specific CD4 T cells in CD8 depleted animals were also detected with similar kinetics and frequencies as seen in control animals (Fig. 6E and 6F). Although SVV-specific CD8 T cells were also detected 7 dpi in CD4 depleted animals, their frequency was initially lower (7 dpi p<0.001 for both CD8 CM and EM, Fig. 6G and 6H). SVV-specific CD8 T cells in CD8 depleted animals were detected 21 dpi (Fig. 6G and 6H), concomitant with the re-appearance of CD8 T cells in these animals (Fig. 1). SVV-specific CD4 T cells were detected 28 dpi in CD4 depleted animals (Fig. 6E and 6F), corresponding with the re-appearance of CD4 T cells in these animals (Fig. 1). As stated above, although the relative percentage of responding T cells within CM and EM subsets in the T cell depleted animals may be comparable to those of control animals, the number of and CD8 and CD4 T cells is still much lower in CD8 and CD4 T cell depleted animals respectively compared to controls.

To determine the magnitude of the frequency of T cells in response to SVV peptide stimulation, we measured the AUC for SVV-specific T cells in all four animals groups between 7 and 35 dpi. This analysis showed that in BAL the magnitude of: (1) SVV-specific CD4 CM T cell response is significantly reduced in CD20 and CD8 depleted animals (p = 0.0155 and p = 0.0036, respectively); (2) SVV-specific CD8 CM response are significantly reduced in CD20 and CD4 depleted animals (p = 0.0146 and p = 0.0023, respectively); and (3) SVV-specific CD8 EM response is significantly reduced in CD4 depleted animals (p = 0.0445) compared to control animals.

### The impact of T and B cell depletion on the development of cytolytic T cells

SVV and VZV infections are associated with the development of cytolytic CD4 and CD8 T cells [46], [52]. In order to determine how loss of lymphocyte subsets alters frequency of T cells with cytolytic potential, we measured the changes in granzyme B (grzmB) expressing CD4 and CD8 T cells (Fig 7, and Table 2). In BAL, we detected an increase in the frequency in grzmB+ CD4 CM 7 dpi in all animals (excluding CD4 depleted animals), which was followed by a quick return to baseline 14 dpi (Fig. 7A). Frequency of grzmB+ CD4 EM increased 3 dpi in control and CD8 depleted animals and 7 dpi in CD20 depleted animals (Fig. 7B). This increase in frequency was sustained until 28 dpi after which frequencies return to baseline levels (Fig. 7B). In CD4 depleted animals, grzmB+ CD4 CM and EM were detected 21 dpi (Fig. 7A and 7B) coinciding with the reappearance of CD4 T cells in these animals (Fig. 1) and peaked in frequency 35 dpi. As described for CD4 T cells, the frequency of grzmB+ CD8 CM T cells increased 7 dpi in all animals (excluding CD8 depleted animals) before returning to baseline 21 dpi (Fig. 7C). However, this increase was significantly reduced in CD4 depleted animals compared to control animals (p <0.001) (Fig. 7C). The frequency of grzmB+ CD8 EM increased 10 dpi in all groups (with the exception of CD8 depleted animals), followed by a small decrease and the establishment of a new set point (Fig. 7D). We detected grzmB+ CD8 CM and EM T cells in CD8 depleted animals 14 dpi, after which the frequency remained stable for the duration of the study (Figs. 7C and D).

A similar pattern of changes in grzmB+ T cell frequencies was observed in PBMCs. We detected a small increase in frequency of grzmB+ CD4 CM 7 dpi in control animals, followed by a return to baseline levels 10 dpi (Fig. 7E). This increase was significantly lower in CD8 and CD20 depleted animals compared to control animals (p <0.001 in both cases) (Fig. 7E). The frequency of grzmB+ CD4 EM T cells increased 10 dpi in CD8 depleted animals and 14 dpi in CD20 depleted and control animals (Fig. 7F), before reaching the same peak magnitude 17 dpi followed by the establishment of a new set point 21 dpi (Fig. 7F). In the CD8 CM subset, the frequency of grzmB+ peaked sharply 7 dpi in all groups, albeit at a significantly lower level in CD4 depleted animals (p<0.001), before returning to baseline levels 10 dpi (Fig. 7G). The frequency of grzmB+ CD8 EM increased 10 dpi in all groups, peaked 17 dpi before establishing a new set point (Fig. 7H). We detected grzmB+ CD8 CM and EM in CD8 depleted animals 14 dpi, which coincides with the re-appearance of CD8 T cells in these animals (Fig. 7H).

Finally, we measured the AUC between 3 and 21 dpi to assess the differences in grzmB expression between the different groups following SVV infection. In BAL, CD20 depleted animals have a significantly reduced frequency of grzmB+ CD4 EM T cells compared to control animals (p = 0.025). In addition, CD4 depleted animals have a significantly reduced frequency of grzmB+ CD8 CM T cells compared to control animals (p = 0.0147). In peripheral blood, the grzmB+ CD4 CM response was lower in both CD8 (p = 0.0265) and CD20 (p = 0.0356) depleted animals. Moreover, the magnitude of grzmB expression in the CD8 CM subset is lower in CD4 depleted animals (p = 0.0002).

## Discussion

Several observations suggest that VZV infection is controlled via cellular immunity rather than humoral immunity. Specifically, children afflicted with T cell deficiencies are at increased risk from progressive varicella. In contrast, children diagnosed with B cell deficiencies such as agammaglobulinemia exhibit uncomplicated varicella [17], [18], [19], [20], [21]. Moreover, whereas VZV antibody administration after contagion but before the appearance of exanthem has been shown to block progression of systemic varicella [53], administration after the onset of varicella has no effect on disease progression [12]. Finally, despite the presence of high VZV-specific IgG titers, aged persons are at increased risk from VZV reactivation than young adults. However, the exact contributions of T cell and B cell immunity to the resolution of acute VZV infection in unvaccinated individuals have not yet been experimentally elucidated. This knowledge would play a critical role in the design of second-generation vaccines against VZV.

SVV is also a neurotropic α herpesvirus and a homologue of VZV that causes varicella in nonhuman primates. We have recently shown that SVV infection of juvenile rhesus macaques recapitulates the hallmarks of primary VZV infection in children including the development of a vesicular rash, the generation of T and B cell responses and the establishment of latency in sensory ganglia [46]. In this model, the animals are infected intrabronchially, which bypasses the early phase of VZV incubation/replication in the head and neck region, resulting in an incubation period of approximately 10 days. Direct inoculation of SVV into the lungs might also result in altered viral amplification since VZV replicates efficiently in tonsilar memory CD4 T cells [48], [49]. Although, this incubation period is slightly shorter than that of VZV (10–21 days), the detection of T and antibody responses in SVV-infected macaques in relation to the onset of the rash occurs with comparable kinetics as those described for VZV [12]. Moreover, whereas natural and experimental SVV infection results in significant morbidity and mortality in other nonhuman primate species, infection of rhesus macaques with SVV results in a milder disease that more closely resembles varicella in humans [54], [55]. These observations suggest that SVV infection of rhesus macaques is a robust model with which to investigate the contribution of CD4 T cell, CD8 T cells and B cell immunity to the control of acute VZV infection.

Our results show that depletion of CD20 B cells and loss of early antibody production does not alter viral loads or disease severity. As expected, CD20-depleted animals were significantly compromised in their ability to generate SVV-specific IgM and IgG antibodies. Our data also suggest that the production of neutralizing antibodies is not required for resolution of varicella as the CD20 animals failed to generate a robust neutralizing antibody response yet had uncomplicated varicella. These data are in agreement with clinical observations that the VZV specific antibody response generated during acute VZV infection contributes little to the immune response to VZV [47]. Indeed, children with congenital agammaglobulinemia experience uncomplicated varicella [56]. Moreover, passive antibody therapy is only successful when administered within the first 72 hours following VZV exposure [24], [25] and has no effect on disease progression if administered after the appearance of the rash [12]. We also observed similar kinetics and magnitude in the frequency of proliferating T cells in BAL and PBMCs of CD20 depleted animals compared to non-depleted controls. Although magnitude and kinetics of T cell proliferation were unchanged in CD20 depleted animals, we detected a lower frequency of SVV-specific T cells 7 and 14 dpi as detected by ICS following peptide stimulation (ORFs 4, 31, 61 and 63) in these animals. One possible explanation for these results is that B cells are an important source of antigen presenting cells in this *in vitro* assay, and their absence results in the detection of a lower frequency of responding T cells. Alternatively the absence of B cells *in vivo* could lead to a change in immunodominance profile, resulting in T cells in CD20 depleted animals preferentially responding to additional ORFs not included in our peptide pools. The SVV-induced increase in frequency of grzmB+ T cells in CD20 depleted animals was similar in kinetics and magnitude to that observed in control animals with the exception that frequency of grzmB+ CD4 CM T cells in PBMC 7 dpi was significantly lower than control animals. This decrease could be due to the fact that B cells contribute to the activation of CD4 T cells in the peripheral blood. Overall, these data are reminiscent of the documented clinical observations and strongly suggest that the development of antibody response during primary SVV/VZV infection is not critical to the resolution of the acute infection. Our data also suggest a potential role for B cells in modulating the specificity of the T cell response, but more conclusive analyses are required to specifically address that question.

Depletion of CD8 T cells resulted in slightly increased viral loads in whole blood and BAL samples but these differences did not reach statistical significance. Interestingly, the time points at which higher viral loads are observed in CD8 depleted animal differ between the BAL and WB, indicative of potentially different mechanisms of control. In the BAL slightly higher viral loads are detected 3 dpi. This is too early to suggest involvement of the CD8 T cell response. Indeed, the antibody that we used for CD8 T cell depletion targets all CD8 + lymphocytes, which in the macaque include NK cells. Thus, it is more likely that the higher viral load detected 3 dpi in CD8 depleted animals is a reflection of NK cell depletion rather than CD8 T cell depletion. In contrast, we observed a higher viral load 10 dpi in WB in CD8 depleted animals, which is more indicative of a defect in the CD8 T cell response. These observations are similar to clinical observations that severe varicella is associated with an absence of NK cells and primed CD8+ T cells responses in children [57]. CD8 depleted animals also experienced a somewhat more prolonged varicella episode and higher number of vesicles. Interestingly, we detected that depletion of CD8 T cells results in an increase in CD4 and B cell proliferation compared to control animals. This compensatory mechanism could have alleviated the consequences associated with loss of CD8 T cell function.

Finally, depletion of CD4 T cells resulted in disseminated varicella, higher viral loads and sustained viremia although complete CD4 T cell depletion was not achieved until 10 dpi. The difference in disease severity between CD4 and CD8 T cell depleted animals cannot be explained by differences in the duration of T cell depletion. In fact, complete CD4 T cell depletion was not achieved until 10 dpi and was very transient in the BAL compared to CD8 T cell depletion and lasted 7 days in PBMC (10–17 dpi), whereas CD8 T cells were depleted for 10 days in BAL and 17 days in PBMC (0–21 dpi). The severe varicella observed in CD4 depleted animals is in line with clinical observations that HIV+ children fail to generate a T cell response and suffer progressive-disseminated varicella as well as additional complications such as varicella pneumonia, hepatitis, coagulopathy and meningoencephalitis [16]. Additionally, HIV patients are more susceptible to herpes zoster during the phase in which absolute numbers of CD4 T cells decline [12], highlighting the importance of CD4 T cell responses in controlling VZV infection. It is possible that the use of anti-CD4 depleting antibody resulted in the depletion of CD4+ macrophages. However, we were not able to detect reduced frequency of monocytes/macrophages in CD4 depleted animals using CD14 and HLA-DR as markers (data not shown). It is also possible that the depleting antibody resulted in the loss of CD4+NK T cells. However, a difference in viral loads between CD4 depleted and control animals is not detected until 7 dpi, indicative of a defect in adaptive rather than innate immunity. Indeed, CD4-depleted animals experienced delayed and reduced CD8 T cell proliferative bursts in both peripheral blood and BAL. This delay correlated with a significant decrease in the frequency of SVV-specific CD8 T cells that were detected by ICS. Moreover, the appearance of SVV-induced grzmB+ CD8 CM T cells was also reduced in these animals. Similarly, B cell proliferation and IgG, IgM and neutralizing antibody production was compromised in CD4 depleted animals. Since data from our CD20 and CD8 depleted animals suggests a minimal role for these lymphocyte subsets in the resolution of acute SVV infection, it is very likely that the severe disease experiences by CD4-depleted animals is a consequence of a compromised CD4 T cell response.

Taken together, data presented in this manuscript suggest that the CD4 T cell response executes unique effector functions that are critical to the resolution of SVV/VZV infection. Although we cannot discount the possibility of synergism between the loss of B and CD8 T cell responses, our own depletion studies and data from additional studies support the hypothesis that CD4 T cells play a unique role during SVV/VZV infection. For instance, in addition to their role in recruiting CD8 CTL to sites of infection via release of Th1 cytokines [12], [58], CD4 T cells can kill VZV-infected cells [31], [59], [60], [61]. Moreover, studies that characterized the VZV-specific T cell response in patients with VZV-induced uveitis revealed that CD4 T cells were responsible for the bulk of the T cell response [62], [63]. VZV-specific CD4 T cells are also preferentially detected following re-exposure to VZV [64]. Taken together, the data presented in this manuscript and from previously published studies strongly suggest that CD4 T cells play a critical role in coordinating the anti-VZV/SVV response during primary infection that goes beyond providing help for CD8 and B cells. Additionally, a recent study investigating CMV-specific immune responses in CMV-seropositive renal transplant patients reported a positive correlation between low CD4 T cell counts and increased CMV DNA in the plasma of patients, suggesting a role for CD4 T cell immunity in controlling CMV reactivation [65]. This report, along with the data presented in this manuscript, suggest that the role of CD4 T cell responses in controlling herpesvirus infections may have previously been under-estimated. More investigations into this topic are needed to uncover the mechanisms by which CD4 T cells participate in the control of herpes viral infections.

In summary, using SVV infection of rhesus macaques as a model of VZV infection [46], we show for the first time *in vivo* the contribution of cellular immunity versus humoral immunity during acute SVV infection. Our results indicate that similar to clinical findings regarding VZV control in children, the ability of young rhesus macaques to control acute SVV infection is mediated by T cell immunity rather than humoral immunity. More importantly, our data strongly suggest that CD4 T cells mediate effector functions that are more important that providing help for antibody or CD8 T cell response in the resolution of acute SVV infection. Future studies will dissect the effector functions of CD4 T cells that are important for protection and elucidate the role of CD4 and CD8 T cell immunity in the protection against SVV reactivation.

## Materials and Methods

### Ethics statement

The study was carried out in strict accordance with the recommendations described in the Guide for the Care and Use of Laboratory Animals of the National Institute of Health, the Office of Animal Welfare and the United States Department of Agriculture. All animal work was approved by the Oregon National Primate Research Center Institutional Animal Care and Use Committee (IACUC protocol # 0779). The ONPRC has been continuously accredited by the American Association for Accreditation of Laboratory Animal Care since 1974 (PHS/OLAW Animal Welfare Assurance # A3304-01). All procedures were carried out under Ketamine anesthesia in the presence of veterinary staff and all efforts were made to minimize animal suffering.

### Cells and virus

SVV was propagated in primary rhesus fibroblasts (1° RF) at 37°C in 175 cm^2^ flasks with DMEM supplemented with 10% FBS. SVV-infected 1° RF were frozen in FetalPlex with 10% DMSO, stored in LN_2_, and assayed by plaque assay. SVV cell lysate was obtained by scraping SVV-infected 1° RF at the height of cytopathic effect (CPE) followed by concentration, and then sonication using 7 pulses of 70–80W (Sonicator 3000, Misonix). The sonicated cell-resuspension was pelleted by centrifugation at 2000rpm for 5 min and frozen at −80°C.

### Animals and sample collection

16 colony-bred Rhesus macaques (*Macaca mulatta,* RM) 3–4 years of age and of Indian origin were used in these studies. They were housed and handled in accordance with the Oregon National Primate Research Center Institutional Animal Care and Use Committee. To deplete CD4 T cells, 4 RM received the humanized monoclonal antibody OKT4-HulgG on −3, 0, and 7 days post-infection (dpi) at a dose of 50mg/Kg. To deplete CD8 T cells, 4 RM received the mouse-human chimeric monoclonal antibody cM-T807 on −3 dpi at a dose of 10mg/Kg, and on 0, 3, and 7 dpi at a dose of 5mg/Kg. To deplete B cells, 4 RM were administered the mouse-human chimeric antibody Rituxan on −7, 0, and 7 dpi at 20mg/Kg. All animals were inoculated intrabronchially with 4×10^5^ PFU SVV as previously described [46]. Blood, bronchial alveolar lavage (BAL), and ∼1 ml saliva samples were collected on days −14, 0, 3, 7, 10, 14, 17, 21, 28, 35, 42, 49, 56, 63, and 70. The absolute numbers of lymphocytes/µL of blood were obtained using a Hemavet (Drew Scientific, Inc., Dallas, TX). BAL samples were pelleted and resuspended in RPMI supplemented with 10%FBS, streptomycin/penicillin, and L-glutamine. Peripheral blood mononuclear cells (PBMCs) and plasma were isolated by centrifugation over a histopaque gradient (Sigma) as per the manufacturer’s recommendation. Saliva samples were centrifuged in order to separate the buccal epithelial cells from the saliva.

### DNA extraction and quantitative PCR

DNA was extracted from heparinized whole blood, BAL cells, saliva and buccal epithelial cells using the Qiagen genomic DNA extraction kit (Qiagen) and SVV DNA loads were determined by real-time PCR using primers and probes specific for ORF21 using the ABI 7700 and ABI StepOne instruments (Applied Biosystems, Foster City, CA) exactly as previously described [46].

### Measurement of T and B cell proliferation

PBMC and BAL cells were surface stained with antibodies against CD8β (Beckman Coulter), CD4 (eBioscience, San Diego, CA), CD28, and CD95 (BioLegend, San Diego, CA) to delineate the naive (CD28+CD95−), central memory (CD28+CD95+), and effector memory (CD28−CD95+) T cell subsets. PBMC and BAL cells were also surface stained with antibodies against CD20 (Beckman Coulter, Brea, CA), CD3 (BD Pharmingen, San Diego, CA), IgD (Southern Biotech), and CD27 (BioLegend) to delineate the naïve (IgD+CD27−), MZ-like (IgD+CD27+), memory (IgD−CD27+) B cell subsets. Cells were fixed and permeabilized according to manufacturer recommendations (Biolegend) before the addition of Ki67-specific antibody (BD Pharmingen). The samples were analyzed using the LSRII instrument (Beckton, Dickinson and Company, San Jose, CA) and FlowJo software (TreeStar, Ashland, OR).

### Granzyme B staining

PBMC and BAL cells were surface stained using antibodies against CD4, CD8β, CD28, and CD95 as described for Ki67 staining. Cells were fixed and permeabilized using fixation buffer (BioLegend), and then stained intracellularly using an antibody against granzyme B (BD Pharmingen). Samples were analyzed using the LSRII instrument and FlowJo software.

### Intracellular cytokine staining

PBMC and BAL cells were either stimulated with SVV lysate (1 ug) for 12 h followed by incubation with Brefeldin A for 6 h, or were incubated with one of the following SVV peptides and Brefeldin A (Sigma, St Louis, MO) for 6 h: open reading frame (ORF) 4; ORF31; ORF61; or ORF63. After stimulation cells were surface stained with antibodies against CD4, CD8β, CD28, and CD95 as described for Ki67 staining. Samples were fixed and permeabilized using fixation buffer (BioLegend), and then stained using antibodies against IFNγ and TNFα (eBioscience). Samples were analyzed using the LSRII instrument and FlowJo software.

### Enzyme-linked Immunosorbent Assay (ELISA)

ELISA plates were coated with SVV lysate overnight at 4°C, washed three times with 0.05% Tween-PBS, and incubated with heat-inactivated (56°C, 30 min) plasma samples in 3-fold dilutions in triplicate for 1 h. After washing three times with 0.05% Tween-PBS, horseradish peroxidase (HRP)-conjugated anti-rhesus IgG (Nordic Immunology, The Netherlands) or anti-rhesus IgM (Brookwood Biomedical, Birmingham, AL) was added for 1 h, followed by addition of *o*-phenylenediamine•2HCl (OPD) substrate (Sigma, St Louis, MO). The reaction was stopped with the addition of 1 M HCl. IgG and IgM endpoint titers were calculated using log-log transformation of the linear portion of the curve, and 0.1 optical density (OD) units as cut-off. IgG and IgM titers were standardized using a positive control sample that was included in every ELISA plate.

### Plaque reduction assay

Neutralizing antibody titers were evaluated as previously described [66] by measuring the plasma dilution at which 50% reduction in SVV plaques was achieved (NT50). Serial two-fold dilutions of heat-inactivated monkey plasma from 0, 14, and 70 dpi were incubated with approximately 150pfu of SVV for 30 minutes at 37°C. Virus/plasma samples were then added to duplicate primary rhesus fibroblast cell monolayers seeded on 12-well plates and incubated for 4 days at 37°C. Monolayers were fixed with methanol and then stained with crystal violet to visualize the SVV plaques.

### Statistical analysis

Repeated measures of ANOVA was used to explore differences between groups. Pair-wise comparisons at each time point were performed using contrast t-test. Statistical significance was determined at the level of 0.05. First order autoregressive covariance structure was used to account for within subject correlation. Due to the small sample size, other complicated covariance analyses was not considered.
